# Supplementary material for: HIVprotI: an integrated web based platform for prediction and design of HIV proteins inhibitors
Source: J Cheminform. 2018 Mar 9;10:12. doi: 10.1186/s13321-018-0266-y (PMC5845081; doi:10.1186/s13321-018-0266-y)
Supplement: Supplementary file 2 — Additional file 2. Source code of HIVProtI web server. [file 13321_2018_266_MOESM2_ESM.zip › HIVprotI_Source-code/hivprotI/weka-3-6-6/doc/weka/attributeSelection/ScatterSearchV1.Subset.html]

ScatterSearchV1.Subset


---


|  |  |  |
| --- | --- | --- |
| **Overview** \| **Package** \| **Class** \| **Tree** \| **Deprecated** \| **Index** \| **Help** \| **Weka's home** | |  |
| **PREV CLASS**   **NEXT CLASS** | **FRAMES**    **NO FRAMES**     **All Classes** |  |
| SUMMARY: NESTED \| FIELD \| CONSTR \| METHOD | DETAIL: FIELD \| CONSTR \| METHOD |  |


---


## weka.attributeSelection Class ScatterSearchV1.Subset

```
java.lang.Object
  weka.attributeSelection.ScatterSearchV1.Subset
```

**All Implemented Interfaces:**: java.io.Serializable

**Enclosing class:**: ScatterSearchV1

---

``` public class ScatterSearchV1.Subset extends java.lang.Object implements java.io.Serializable ```

**See Also:**: Serialized Form

---

**Constructor Summary**

`ScatterSearchV1.Subset(java.util.BitSet subset,
double merit)`


| **Method Summary** | |
| --- | --- |
| `ScatterSearchV1.Subset` | `clone()` |
| `boolean` | `isEqual(ScatterSearchV1.Subset othersubset)` |

**Methods inherited from class java.lang.Object**

`equals, getClass, hashCode, notify, notifyAll, toString, wait, wait, wait`

**Constructor Detail**

### ScatterSearchV1.Subset

```
public ScatterSearchV1.Subset(java.util.BitSet subset,
                              double merit)
```


**Method Detail**

### isEqual

```
public boolean isEqual(ScatterSearchV1.Subset othersubset)
```

---


### clone

```
public ScatterSearchV1.Subset clone()
```

:   **Overrides:**: `clone` in class `java.lang.Object`


---


|  |  |  |
| --- | --- | --- |
| **Overview** \| **Package** \| **Class** \| **Tree** \| **Deprecated** \| **Index** \| **Help** \| **Weka's home** | |  |
| **PREV CLASS**   **NEXT CLASS** | **FRAMES**    **NO FRAMES**     **All Classes** |  |
| SUMMARY: NESTED \| FIELD \| CONSTR \| METHOD | DETAIL: FIELD \| CONSTR \| METHOD |  |


---
